# Phenolic Compounds of Therapeutic Interest in Neuroprotection

**DOI:** 10.3390/jox14010014

**Published:** 2024-02-06

**Authors:** José Manuel Nájera-Maldonado, Ricardo Salazar, Patricia Alvarez-Fitz, Macdiel Acevedo-Quiroz, Eugenia Flores-Alfaro, Daniel Hernández-Sotelo, Mónica Espinoza-Rojo, Mónica Ramírez

**Affiliations:** 1 Faculty of Chemical Biological Sciences, Autonomous University of Guerrero, Chilpancingo 39087, Mexico; 09118496@uagro.mx (J.M.N.-M.); eugeniaflores@uagro.mx (E.F.-A.); dhernandez@uagro.mx (D.H.-S.); monicaespinoza@uagro.mx (M.E.-R.); 2 CONAHCYT National Council of Humanities, Sciences and Technologies, Autonomous University of Guerrero, Chilpancingo 39087, Mexico; rsalazarlo@conahcyt.mx (R.S.); palvarezfi@conahcyt.mx (P.A.-F.); 3 National Technological Institute of Mexico, Technological/IT Institute of Zacatepec, Zacatepec 62780, Mexico; macdiel.aq@zacatepec.tecnm.mx

**Keywords:** neurodegenerative diseases, alternative treatments, phenolic compounds, antioxidants, molecular mechanisms

## Abstract

The number of elderly people is projected to double in the next 50 years worldwide, resulting in an increased prevalence of neurodegenerative diseases. Aging causes changes in brain tissue homeostasis, thus contributing to the development of neurodegenerative disorders. Current treatments are not entirely effective, so alternative treatments or adjuvant agents are being actively sought. Antioxidant properties of phenolic compounds are of particular interest for neurodegenerative diseases whose psychopathological mechanisms strongly rely on oxidative stress at the brain level. Moreover, phenolic compounds display other advantages such as the permeability of the blood–brain barrier (BBB) and the interesting molecular mechanisms that we reviewed in this work. We began by briefly outlining the physiopathology of neurodegenerative diseases to understand the mechanisms that result in irreversible brain damage, then we provided an overall classification of the phenolic compounds that would be addressed later. We reviewed in vitro and in vivo studies, as well as some clinical trials in which neuroprotective mechanisms were demonstrated in models of different neurodegenerative diseases such as amyotrophic lateral sclerosis (ALS), Alzheimer’s disease (AD), Parkinson’s disease (PD), ischemia, and traumatic brain injury (TBI).

## 1. Introduction

Worldwide, the number of elderly people will double in the next 35 years, leading to an increase in the incidence of neurodegenerative disorders [1]. Neurodegenerative diseases are chronic disorders of the central nervous system, such as Alzheimer’s disease (AD), Parkinson’s disease (PD), dementia with Lewy bodies (DLB), multiple system atrophy (MSA), progressive supranuclear palsy (PSP), and Huntington’s disease (HD) [2]. One of the main causes of neurodegeneration is aging, which can lead to changes in the brain related to its tissue homeostasis and contribute to the development of neurodegenerative diseases. Some neurodegenerative diseases share physiopathology characteristics, such as aggregates of misfolded proteins and the formation of extracellular or intracellular plaques [2,3]. It has been observed that severe acute respiratory syndrome coronavirus 2 (SARS-CoV-2) could exacerbate neurodegenerative diseases as it infects the brain by binding to angiotensin-converting enzyme 2 (ACE2) receptors that are widely expressed in the brain, including on dopaminergic neurons, which, along with consequent neuroinflammation, contribute in the short and long term to the onset of disorders such as PD [4]. Furthermore, comorbidities such as diabetes and hypertension can promote these processes. In cells, mitochondria play a key role in oxidative phosphorylation during the catabolism of glucose and the generation of cellular energy. Nevertheless, they lead to the formation of reactive oxygen species (ROS) due to various alterations in the antioxidant system that oxidizes molecules, including lipids, proteins, nucleic acids (DNA/RNA), and enzymes [5]. Lipids are essential in neuronal function, acting as a barrier between the intracellular and extracellular space. They are prone to oxidation by free radicals, which alter cell permeability and facilitate the entry of molecules that cannot normally enter except through specific channels (K^+^, Ca^2+^). They might damage proteins such as enzymes or receptors, altering cellular functioning. These alterations are present in AD, PD, and HD [6]. Standard treatment for neurodegenerative diseases does not work for most patients, who require personalized treatment. Research with new drugs has not obtained favorable results, which raises the need for new therapeutic schemes that could include phenolic compounds. It has been observed that, when consumed, phenolic compounds may function as natural inhibitors of enzymes involved in glucose metabolism, particularly α-glucosidase and α-amylase [7]. Additionally, several phenolic compounds can cross the blood–brain barrier (BBB) and have important biological effects in in vitro and in vivo models of neurodegenerative diseases, which are mainly associated with their antioxidant and anti-inflammatory capacity [8] and can even improve permeability when there are BBB alterations [9].

We reviewed information on phenolic compounds that have shown a neuroprotective effect in vitro or in vivo regardless of the year of publication. Other important selection criteria for this work were that the authors evaluated isolated and purified compounds. Thus, the information could be extrapolated to future research and even clinical trials to test their efficacy and safety. We included both simple phenolic compounds and polyphenols if they met the criteria described above. Finally, we included curcumin, a well-studied compound with anti-neurodegenerative activity, to outline and compare the information that we collected. When possible, we included clinical trials.

## 2. Physiopathology of Neurodegenerative Diseases

One of the main characteristics of neurodegenerative diseases is the imbalance and increase in reactive oxygen and nitrogen species (RNS), which can occur due to aggregates of altered proteins [10] or altered levels of neurotransmitters, such as glutamate. Glutamate is the most important excitatory neurotransmitter in mammals, as it is involved in cognitive processes, memory, synaptic plasticity, and neuronal development [11]. Mutations in genes encoding ionotropic glutamate receptor subunits, which are critical for synaptic transmission and plasticity, are associated with intellectual disability, autism spectrum disorders, and neurodegenerative diseases. In contrast, mutations in metabotropic glutamate receptors (GluRs), which play a role in modulating neural transmission, are preferentially associated with psychiatric or neurodegenerative disorders. The metabotropic receptors mGluR1 and mGluR5 Group I are coupled to the activation of Gq/11 proteins, which couple to signaling pathways that can affect protein kinase activation and stimulate Ca^2+^ release from neuronal stores (endoplasmic reticulum), triggering cell death processes [12].

Glutamate concentrations at the intracellular level are in the millimolar range, while at the extracellular level, they are at micromolar concentrations. Excess glutamate causes neuroinflammation and excitotoxicity in vitro and in vivo by the overactivation of ionotropic receptors and, as a result, contributes to neuronal death by apoptosis and autophagy [13].

Glutamate can reduce the cell survival rate by up to 60% compared to the control group in mouse hippocampal neuronal HT22 cells. Furthermore, glutamate treatment increases ROS levels [14]. The increase in ROS is due to increased Ca^2+^, as it can elevate mitochondrial overload, nitric oxide production, lipid peroxidation, and cytochrome C dissociation [15,16]. The use of phenolic compounds has been proposed to reduce oxidative stress and mitigate some of the alterations generated by the deregulation of the redox system since the compounds can eliminate superoxide, peroxide, singlet oxygen, hydroxyl radical, nitric oxide (NO), and peroxynitrite (OONO-), which are constantly produced in cells because of cellular respiration [17,18].

Damage to cell populations of astrocytes and neurons leads to the release of glutamate into the extracellular space, exacerbating neurodegenerative pathologies. Currently, there are a few drugs approved for the treatment of neurodegenerative diseases. For example, in the handling of AD, there are three classes of approved medications, cholinesterase enzyme inhibitors, and N-methyl D-aspartate (NMDA) antagonists, which only alleviate the symptoms of AD but do not cure or prevent the disease [19]. In January 2023, the FDA approved a new treatment with a human anti-beta amyloid (Aβ) monoclonal antibody, which is effective in reducing Aβ accumulation and slowing cognitive decline [20].

**Figure 1:**
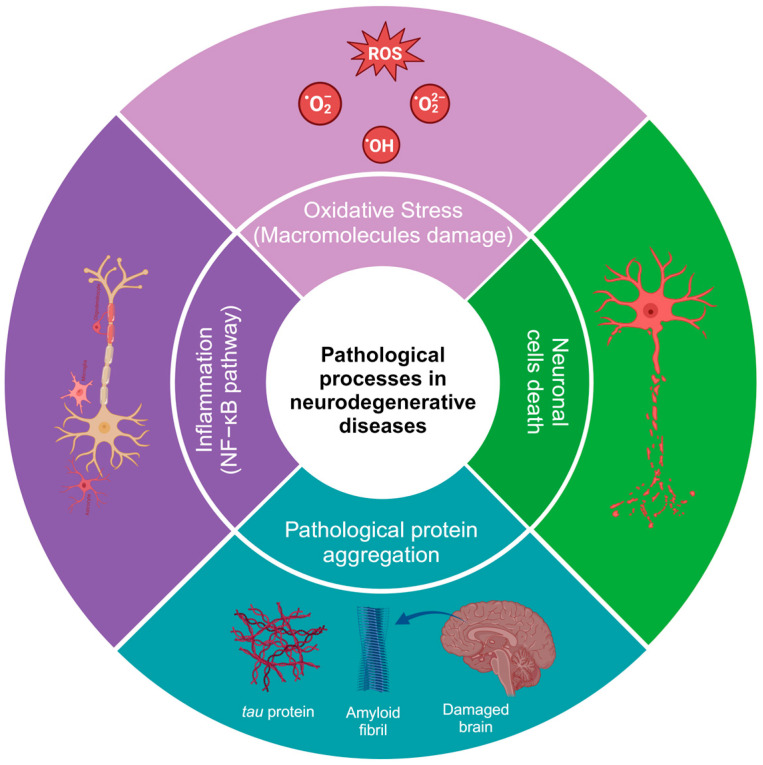
Pathological processes in neurodegenerative diseases. The image was created in BioRender (www.biorender.com, accessed on 25 January 2024).

HD is caused by the expression of the mutant huntingtin protein, and these proteins can interact with hydrogen peroxide, glutathione, and, especially, copper, which promotes the oxidation of the N171 huntingtin fragment and leads to its oligomerization (there is an average of one copper for every three molecules of the huntingtin N171 protein) [21]. Additionally, in HD, alterations can be found in neuronal myelination, leading to attempted remyelination by oligodendrocytes. Iron is critical for oligodendrocyte differentiation and proliferation processes and is dependent on high iron stores. Studies in HD patients and healthy patients have shown an increase in iron in certain brain regions, such as the striatum [22]. In neurodegenerative diseases, inflammation is caused by protein aggregation or by the effect of autoimmunity, leading to an increase in cytokines, chemokines, and ROS [23,24]. Some of the most common alterations in the physiopathology of neurodegenerative diseases are shown in Figure 1. It has been demonstrated that natural and synthetic phenolic compounds can scavenge free radicals and chelate transition metals, halting progressive oxidative damage [25].

## 3. General Aspects of Phenolic Compounds

Phenolic compounds are natural metabolites that consist of at least one aromatic ring (benzene) with one or more hydroxyl substituents. They can be as simple as pyrogallol or as complex as tannic acid. The main classification criteria for phenolic compounds rely on (a) the number of rings and (b) the substitution patterns of the ring [26]. Based on the number of rings, phenolic compounds are classified as simple phenols, which can have one (single phenols) to two rings (biphenols), and polyphenols (>2 rings) [27].

**Figure 2:**
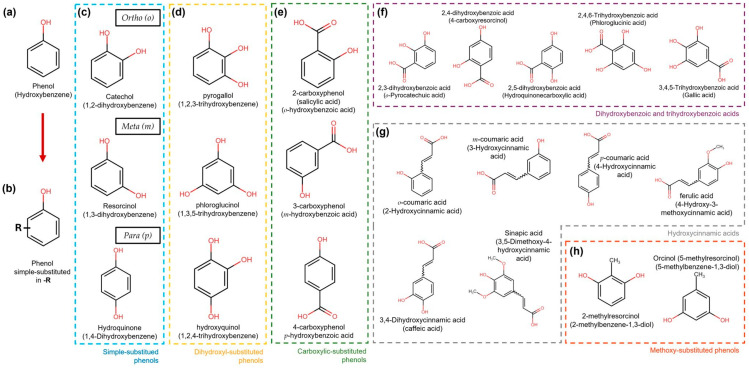
Simple phenol compound configurations and examples. (**a**) Phenol structure. (**b**) Simple phenolic compounds are substituted in “-R” by an organic, hydroxy, carboxy, or other functional group. (**c**) In simple substituted phenol compounds, also named hydroxyphenols or dihydroxybenzenes (blue dotted box), the “-R” group can be in the ortho (*o*), meta (*m*), or para (*p*) position of the ring. (**d**) Dihydroxyphenols (trihydroxybenzenes) (yellow dotted box). (**e**) Hydroxybenzoic acids are carboxylic-substituted phenols in o-, m- or p-positions (green dotted box). (**f**) Dihydroxybenzoic and trihydroxybenzoic acids are shown in the purple dotted box. (**g**) Phenols with the carboxylic acid functional group separated from the ring by a C=C bond are known as hydroxycinnamic acids (grey dotted box). (**h**) Simple phenols also can be substituted with methoxy groups (orange dotted box).

Simple phenolic compounds have a C_6_ skeleton, which was substituted in the “-R” group. The last can be an organic, hydroxy, carboxy, or other functional group (Figure 2). Regarding the substitution patterns to “-R”, the configurations “ortho”, “meta”, and “para” in single phenols refer to 1,2, 1,3, and 1,4 substitution patterns by two hydroxyl functional groups in the benzene ring, respectively (Figure 2) [28]. However, benzenes can also be substituted with two or three functional groups. Some examples of these patterns are more extensively illustrated in Figure 2.

**Figure 3:**
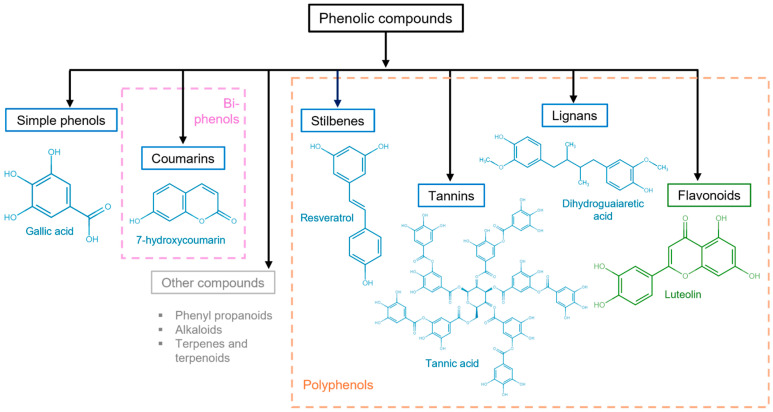
Polyphenolic compound classification and examples. Phenolic compounds are classified into simple phenols (see Figure 2), biphenols (pink dotted box), or polyphenols (orange dotted box). Their principal categories are phenolic acids (benzoic acids, cinnamic acids, and phenylacetic acids), coumarins, stilbenes, tannins (condensed tannins, hydrolyzable tannins, and complex tannins), and lignans, which are known as non-flavonoids (blue solid box) and flavonoids (antoxanthins and anthocyanidins) (green solid box). Other polyphenolic compounds (gray solid box).

Other important criteria to classify phenolic compounds are the arrangement of the rings in polyphenols and the number and type of substituents to those rings. This classification yields flavonoids, phenolic acids, tannins, stilbenes, and lignans (Figure 3) [29,30].

It is generally known that phenolic compounds display a wide spectrum of bioactivities; one of the most relevant and extensively studied is antioxidant bioactivity. The antioxidant capacity of phenolic compounds is due to their reducing property acting as hydrogen or electron donors. Furthermore, the number of hydroxyl groups and their positions relative to the carboxyl functional group influence their antioxidant capacity [31,32,33]. Antioxidant properties of phenolic compounds are of particular interest for neurodegenerative diseases whose psychopathological mechanisms strongly rely on oxidative stress at the brain level. Antioxidant bioactivities are displayed from simple phenols to the most complex ones, as will be described below.

In vitro, pyrogallol (Figure 2d) has been shown to inhibit alpha-synuclein (αSyn), a protein related to neurodegenerative diseases, such as PD, DLB, and MSA, by interacting with the N-terminal and NAC (NAM, ATAF1/2, and CUC2) domains of the protein [34]. Tyrosol has been shown to prevent the formation of αSyn aggregates, a protein implicated in oxidative stress and neurotoxicity [35]. In a different study, using a mouse model, phloroglucinol (Figure 2d) showed permeability to BBB, which also inhibited the formation of αSyn and β-amyloid (Aβ) peptide aggregates, arrested cognitive impairment, and prevented the cytotoxicity of both αSyn and β-amyloid; a molecular docking model showed hydrophobic interactions and hydrogen bonds between phloroglucinol and Aβ1-42/α-syn [36].

From polyphenolic compounds, flavonoids are the major group and are widely found in fruits, generally in the form of secondary metabolites [37]. The structure of flavonoids consists of two benzene rings (A and B) linked to a third ring (C), a heterocyclic pyran, or pyrone [31]. Flavonoids are subdivided into two classes: those containing a hydroxyl group on C-3 of the C ring, known as hydroxy flavonoids, and flavonoids lacking the hydroxyl group, known as 3-deoxyflavonoids. The hydroxyl groups provide flavonoids with a great antioxidant capacity. Thus, they can act as reducing agents, hydrogen donors, scavengers of superoxide radicals, and singlet oxygen. Flavonoids can even activate antioxidant enzymes [38].

Phenolic compounds, such as tannins, can interact with proteins, ROS/RNS, and transition metals due to the inherent chemical and structural properties of the compound and proteins; interactions with proteins can be hydrophobic between the aromatic ring of the tannin and the hydrophobic region of the protein, in addition to the formation of hydrogen bonds. Interactions may vary in the size and charge of the molecule or protein, as well as the length and flexibility of the compound [39]. Thus, they illustrate a potential to be used to inhibit the formation of protein aggregates in neurodegenerative diseases.

## 4. Molecular Mechanisms of Phenols to Prevent Neurodegeneration

Treatment with phenolic compounds can modulate transition metal-related events in the pathophysiological processes of neurodegenerative diseases and regulate the production of ROS through different mechanisms. In HD, there are alterations in neuronal myelination, leading to an attempt at remyelination by oligodendrocytes, and iron is essential for the processes of differentiation and proliferation of oligodendrocytes, which depend on high iron stores. Natural and synthetic phenolic compounds can scavenge free radicals and chelate transition metals, halting progressive oxidative damage [21,22]. Polyphenol ligands can strongly stabilize Fe^3+^ compared to Fe^2+^. For example, in the presence of O_2_, the Fe^2+^ complexes of catecholate and gallate were rapidly oxidized to form Fe^3+^-polyphenol complexes [40].

Another alternative is the formation of complexes within the carbonyl group; metal ion complexes are usually formed on the C-4 keto group and the hydroxyl group on the C-5, leading to the formation of metal–flavonoid complexes. When the carbonyl group is absent, the chelation capacity of the compounds depends on the hydroxylation pattern, as the compounds tend to serve as hydrogen bridge donors [41]. The antioxidant containing the H atom reacts with the free radicals, and the free radical is stabilized to form a neutral species, while the antioxidant becomes a free radical species. The phenolic antioxidant (PA) can provide an H atom to the free radical and produce a nonradical substrate species (RH, ROH, or ROOH) and an antioxidant free radical [40].

Phenol oxidation results in the formation of quinone products of its antioxidant, and quinones can form reaction adducts of Michael addition in nucleophilic groups such as thiols and amines (Gly, Nα-acetyl-Lys, Nε-acetyl-Lys, and L-Lys) from some proteins, such as albumin [42].

Moreover, phenolic compounds can interact with proteins through noncovalent bonds comprising hydrogen bonds, hydrophobic bridges, van der Waals forces, and ionic interactions with proteins, such as with proline-rich proteins, albumin, gelatin, b-lactoglobulin, lysozyme, and serum proteins. Oxidation products of phenolic compounds such as quinones can form covalent bonds by binding to amino groups or amino acid side chains [43]. These interactions could explain why they can modulate signaling pathways involved in neurodegenerative processes and have a positive or negative effect, depending on the proteins with which they interact.

## 5. Metabolism of Phenol Compounds

Phenolic compounds are obtained from the consumption of fruits and vegetables, and the intestine is the first physiological barrier they cross to enter the circulation and distribute to different organs. In healthy individuals and ileostomy patients who ingested beverages containing dietary (poly) phenols derived from green tea, apples, grapes, and citrus fruits, catechins, and gallocatechin were detected in ileal fluid and urine and were found in similar amounts in both groups, indicating that their absorption was in the small intestine [44].

**Figure 4:**
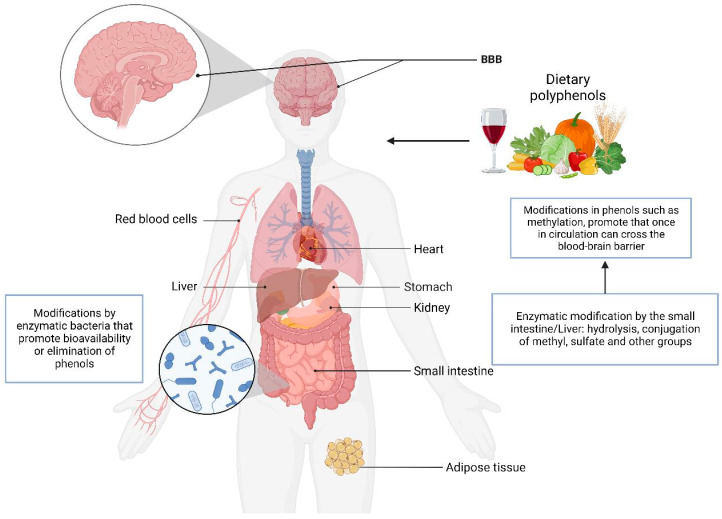
Organs involved in metabolism and phenolic compound bioavailability. BBB (blood–brain barrier). After ingesting phenols, they reach the stomach and can be modified or degraded by the pH. Phenols are absorbed in the small intestine and distributed through the bloodstream to various organs and tissues. Phenols can be broken down or modified by the intestinal microbiome and reabsorbed in the colon. The liver metabolizes drugs and phenols through hepatic enzymes. The BBB is highly selective and does not allow for the entry of polar molecules. However, additions of functional groups such as methyl groups allow them to cross the BBB. It is also proposed that there are transporters for phenolic compounds. Compounds can be excreted in urine or feces. The image was created in BioRender (www.biorender.com, accessed on 25 January 2024).

Of the polyphenols obtained from fruit intake, less than 10% are absorbed, and the rest reach the colon and are metabolized by intestinal microorganisms to low-molecular-weight metabolites. The biotransformation of the compounds includes hydroxylation, oxidation, decarboxylation, methylation, isomerization, hydration, dehydrogenation, and glycosylation. The methylation of phenols promotes absorption and hepatic metabolic stabilization, increasing the phenols’ bioavailability [45]. The bioavailability of phenolic compounds may vary depending on the pH, which could favor absorption in the intestine. During gastrointestinal digestion, Phase II metabolic processes (glucuronidation, sulfation, and methylation) and enzymatic and microbial catabolism modify the compounds [46]. Extrinsic and intrinsic factors, such as exercise, diet, and stress, can alter the progression of neurodegenerative diseases and gut microbiota [47]. This suggests that the chemical and enzymatic activities of both humans and the intestinal microbiota alter the catabolism processes of phenols, as well as their bioavailability, which poses a pharmacological challenge for the utilization of the compounds. However, they have shown significant antioxidant and anti-inflammatory activity and protein aggregate inhibitory activity. Figure 4 shows the organs involved in the metabolism of phenolic compounds.

Another physiologic barrier is the BBB, which has the function of regulating the entry of molecules necessary for brain function, maintaining a stable brain environment by protecting it from foreign substances, establishing a cellular barrier formed through transporters and a barrier with enzymes such as gamma-glutamyl transferase (GGT) and alkaline phosphatase (ALP) that prevent the entry of substances not required by the brain [48].

A pathway that could be involved in the metabolism of phenolic compounds is that of enzymes that modify the compounds and favor their entry into the brain, such as arylsulfatase (ARS), glutathione S-transferase (GST), gamma-glutamyl transferase (GGT), N-acetyltransferase (NAT), catechol O-methyl transferase (COMT), and UDP-glucuronosyltransferase (UGT). In vitro, the transport of phenolic compounds was evaluated through cerebral microvascular endothelial cells, and there was differential transport depending on the chemical modifications in the compounds. Those that included the addition of methyl and sulfate groups (for example, 4-methyl catechol *O*-sulfate) had better transport and biological effects [49]. The blood–brain barrier is one of the major limitations of drugs in development. However, studies have revealed in situ and in vitro BBB models evaluating the transport of the flavonoids naringenin and quercetin, showing that both are permeable to the BBB in situ [50]. These tests highlight the therapeutic potential of phenolic compounds since modifications in the structure of the compounds facilitate their entry into the brain.

One of the main limitations is that the pharmacodynamics and pharmacokinetics of phenols are not yet fully understood. However, there is already evidence of the possible mechanisms of phenols. A common example is curcumin. It is one of the most studied phenols and has a more described metabolism in animal models. Curcumin has been found in low concentrations in the liver, kidney, and plasma [51]. After oral ingestion, curcumin is absorbed into the intestine and enters the bloodstream. At pH > 7, curcumin degrades to trans-6-(40-hydroxy-30-methoxyphenyl)-2, 4-dioxo-5-hexanal, ferulic acid, feru-loylmethane, and vanillin within 30 min. Under acidic conditions, the degradation of curcumin is slower; 20% is degraded in about 1 h. Therefore, liposomes, nanoparticles and polymeric micelles, phospholipid complexes, and microemulsions [52] have been designed to improve the bioavailability of curcumin and could also be applied to the various phenols mentioned in this review.

In a study performed in male KM mice, using liquid chromatography–mass spectrometry in the tissues of mice after intravenous administration, curcumin and one of its metabolites, tetrahydrocurcumin, were found in the liver, while curcumin and dihydrocurcumin were detected in the kidney. Only curcumin was found in the brain, not its metabolites [53]. In addition, it has been reported that bacteria (*E. coli*) isolated from two healthy patients can reduce curcumin into two intermediate products: dihydro-curcumin, and then into the final product tetrahydrocurcumin by an NADPH-dependent enzyme [54]. However, a study with stable polymeric nanoparticles (nano curcumin) showed improved oral absorption in mice since it required 20 times less to obtain the same concentration in plasma and central nervous system tissue than the oral consumption method without nanoparticles [55].

Currently, in silico analyses of phenolic compounds have gained great relevance due to the information they provide, such as possible molecular targets, the type of interaction with the molecules, pharmacological kinetics, strength, and the kinds of interaction with molecules, as well as the low cost and time needed for the discovery of new potential drugs [56,57]. There are still many limitations, one of the main ones being the lack of high-resolution structures of membrane transporters. The transport of phenolic compounds is limited by the polarity of the molecules, the number/type of bonds, and the molecules with which they interact, among other molecular characteristics. It has been observed that modifications in compounds, such as methylation, could promote passive diffusion or transport across the membrane [49]. Phenol transporters are not fully elucidated, and in silico analyses of phenolic compounds have shown that the bilitranslocase (BTL) transporter, a widely distributed epithelial transporter of the stomach, intestine, kidney, and liver, could be a potential phenol transporter. From a database of 300 compounds, 205 showed the potential to interact with BTL, suggesting that it is a phenol transporter. However, experimental evidence is still lacking to confirm these findings [58].

On the other hand, Adbelkarim et al. [59] performed an in-silico analysis of phenolic compounds derived from *Moroccan Satureja nepeta*, such as quercetin, catechin, and gallic acid, which have a high affinity for the IL-6 receptor, an interleukin that is related to inflammation, apoptosis, and cell differentiation. In addition, it has been shown to have an important role in neurodegenerative diseases [60,61,62].

In a pathway-enrichment analysis of genes differentially expressed in LUHMES cells treated with low molecular weight phenols, it was identified that phenols regulate signaling pathways related to apoptosis, cell death, cell homeostasis, cell replication, and cell development processes [63]. These data show the potential of the compounds to interact with or favor signaling pathways involved in neuroprotection.

## 6. Effect of Phenolic Compounds In Vitro in Models of Neurodegeneration

Studies in models of neuronal damage by excitotoxicity induced by NMDA receptor overactivation showed that treatment with phenolic compounds such as mangiferin and morin promoted neuroprotection through the activation of the antioxidant enzyme system, reducing ROS formation and restoring mitochondrial membrane potential. In addition, they regulate the protein kinase B (AKT) and extracellular signal-regulated kinase (ERK) 1/2 pathways related to neuronal survival, as well as cytosolic levels of Bax and NF-κB translocation, which individually and/or together reduce neuronal death and inflammation [64]. These findings demonstrate that phenolic compounds can modulate neuronal apoptosis by regulating proteins involved in apoptosis (Bax), inflammation (factor nuclear kappa-light-chain-enhancer of activated B cells (NF-κB), and cell survival, such as AKT and ERK 1/2.

In addition, phenolic compounds have been shown to modulate inflammation, such as catechol-O-sulfate, pyrogallol-O-sulfate2, 1-O-methylpyrogallol-O-sulfate3, 4-O-methylgallic acid-3-O-sulfate, 2-O-methylpyrogallol-1-O-sulfate, vanillic acid 4-O-sulfate, 4-methylcatechol O-sulfate4, and 4-O-methylgallic acid, which cross the BBB in an immortalized human brain microvascular endothelial cell (HBMEC) line, showing that phenols can cross the BBB in vitro differently for each compound according to their chemical structure, particularly for gallic acid derivatives; the combination of methylation and sulfation increases their ability to cross the BBB. The anti-inflammatory capacity of phenols is due to their ability to inhibit the translocation of NF-ĸB, which is a transcription factor regulator of inflammation. Simultaneously, the phenols contributed to an antioxidant effect by decreasing ROS and RNS [49]. Some phenolic compounds are shown in Figure 2 and Figure 3.

In oligodendrocytes and neurons, morin decreased the levels of ROS and rescued the loss of membrane potential due to the overactivation of α-amino-3-hydroxy-5-methyl-4-isoxazolepropionic acid (AMPA) receptors, which was related to cell protection. Mangiferin did not decrease the levels of ROS or rescue the loss of mitochondrial membrane potential due to the overactivation of AMPA receptors. However, attenuated Ca^2+^ overload, which could explain a neuroprotective effect not related to the decrease in ROS, could be an alternative mechanism of some phenolic compounds to decrease neuronal cell death in conditions of excitotoxicity not generated by oxidative stress, in addition to being a possible additive mechanism in conditions of oxidative stress [65]. In the primary culture of neurons treated with two phenolic compounds (mangiferin and morin), the levels of AKT and ERK 1/2, cytosolic BAX, and NFKB increased, and such modulation contributed to a reduction in neuronal death [64].

In another study conducted to analyze the effects of resveratrol on monocyte-derived dendritic cells (DCs), resveratrol was revealed to induce immune tolerance in DCs during cell differentiation and activation and strongly downregulate the expression of the costimulatory molecules CD40, CD80, and CD86. Furthermore, resveratrol regulates the secretion of the cytokines IL-10 and IL-12, which are associated with the effect of resveratrol on multiple targets, such as NF-ĸB, which subsequently induces a transcriptional program that results in the activation of regulatory T cells [66]. The effect of phenolic compounds on cell differentiation has also been evaluated in the microglial cell line BV2, which was exposed to lipopolysaccharide, a potent inducer of inflammation, and when exposed to curcumin treatment, microglial activation was inhibited. Curcumin treatment resulted in changes in microglia from the pro-inflammatory M1 phenotype to the anti-inflammatory M2 phenotype by decreasing the expression of M1 markers (iNOS, IL-1β, IL-6, and CD16/32) and elevating the expression of M2 markers (IL-4, IL-10, and CD206), as well as attenuating the activation of the Toll-like receptor-4 (TLR4)/NF-κB pathway [67]. These data suggest that phenolic compounds may influence neurodegenerative diseases by decreasing inflammation by inhibiting transcription factors related to neuroinflammation. Simple compounds (with only one aromatic ring), such as resorcinol [68,69], phloroglucinol [70,71], and pyrogallol [72,73], among others, have shown antioxidant and anti-inflammatory properties, as well as the modulation of signaling pathways. For this reason, they could be used as therapeutic agents in neurodegenerative diseases.

Low-molecular-weight phenolic compounds, such as catechol-O-sulfate (cat-sulf) and pyrogallol-O-sulfate (pyr-sulf), found in plasma following the ingestion of polyphenol-rich foods have been shown to have beneficial effects by reducing oxidative stress and neuroinflammation and modifying cell death mechanisms. In a 3D model of human Parkinson’s disease cells, phenol treatment promoted the expression of target genes of the oxidative stress pathway (FTH1), apoptosis (AKT1, BCL2L1), autophagy (ATG5, ATG12, BECN1), or the unfolded protein response (UPR) (ATF4, ATF6, DDIT3, CALR, HSPA4, HERPUD1). In addition, phenols regulate cellular senescence-related pathways, such as interleukin-7 (IL-7), the Janus kinase/signal transducer and activator of transcription (JAK/STAT), phosphatidylinositol 3-kinase (PI3K/AKT), and the peroxisome proliferator-activated receptor alpha (PPAR-α) [63].

Microglial cells regulate the inflammatory cellular response, and the overactivation of microglia promotes the release of pro-inflammatory mediators that are potentially neurotoxic, in addition to the production of ROS that promotes neuronal cell death. In BV-2 (murine microglial cells) and SH-SY5Y (human neuroblastoma) cells, the use of maple syrup enriched with phenols decreased the levels of ROS, NOS, and inflammatory mediators such as IL-6, prostaglandin E2 (PGE 2), and tumor necrosis factor α (TNF-α) [62].

## 7. Effect of Phenolic Compounds in Models of Neurodegeneration In Vivo

A loss of neuronal populations characterizes neurodegenerative processes. During these events, the process of apoptosis plays a significant role in the oxidative process since, during the process of intrinsic apoptosis, there is an uncoupling of the electron chain and the promotion of oxidative phosphorylation. There is a decrease in ATP synthesis, as well as the accumulation of ROS. On the other hand, mitochondrial dysfunction promotes ROS accumulation, which can damage DNA and promote cell death [74]. In an ischemia model using Wistar rats, the administration of a synthetic antioxidant (di-tert-butyl-bisphenol) before ischemic injury decreased the levels of hypoxia-inducible factor 1α and glucose transporter 1, allowing for the maintenance of ATP concentration in neuronal tissue. Additionally, the inhibition of proapoptotic factors improved brain tissue viability after injury [75].

Mice (5XFAD) that developed severe amyloid pathology due to the accumulation of elevated levels of Aβ42 protein were treated with 7,8-dihydroxyflavone. An improvement in the cognitive ability of mice was observed when challenged in a Y maze that assesses spatial working memory function. In this regard, tyrosine receptor kinase B (TRKB) signaling, a pathway implicated in AD pathogenesis, was restored in mice [76]. In another study, using the animal model 5XFAD, the compound ortho-catechol inhibited the formation of protein aggregates due to the characteristics of the polyhydroxy flavonoid, such as the structural rigidity of the phenol and its ability to form H-bonds that prevent the formation of Aβ aggregates through its direct binding and destabilization of the aggregates. Likewise, this flavonoid can inhibit activity on tau (τ) protein aggregates. Aβ and τ are two proteins in developing neurodegenerative diseases [77].

An important event in neurodegeneration is the shift of microglia to a pro-inflammatory profile. In a chronic neuro-inflammatory model, glial fibrillary acidic protein-interleukin 6 (GFAP-IL6) in male and female Wistar mice treated with apigenin reduced histological inflammatory markers (Iba-^1+^), and after chronic treatment with apigenin, the number of Iba-^1+^ cells decreased [78]. This result is consistent with those mentioned above, in which it has been observed that phenolic compounds, in addition to reducing inflammation and ROS, can also modulate the differentiation of microglia or immune system cells toward anti-inflammatory profiles.

## 8. Effect of Phenolic Compounds on Neurodegeneration in Clinical Trials

There are enzymes in charge of regulating ROS at the cellular level. One example is superoxide dismutase (SOD), which is a group of metalloenzymes that provides an important antioxidant defense mechanism [79]. The antioxidant capacity of phenolic compounds is due to the capacity of the OH groups in these molecules to yield protons and electrons to reduce the oxidation caused by ROS, in addition to suppressing or delaying the autoxidation of organic molecules [80]. This makes them candidates for alternative therapies in which oxidative stress is one of the main drivers of cell damage.

ROS arises from molecular oxygen because of one-electron reduction reactions. The one-electron addition process to molecular oxygen results in the formation of superoxide anions, and the two-electron reduction of oxygen produces hydrogen peroxide [81]. In neurodegenerative diseases, ROS promotes the formation of protein aggregates. In AD, amyloid plaques are formed because of β- and γ-secretase enzymatic activity, and Aβ peptides aggregate oligomerically, conferring oxidative damage to neurons and glial cells through excitotoxicity events that have been related to their interaction with receptors such as NMDA receptors [82]. Compounds such as catechins have antioxidant and antiradical effects [61,83], are hydrogen donors, and inhibit ascorbate-induced lipid peroxidation in brain mitochondria. This effect is attributed to the ortho-3V, 4Vdihydroxy moiety in the B-ring of the molecule, which is involved in electron delocalization and radical stabilization [84]. Catechin shows antioxidant (reducing) activity mainly in two of its three aromatic rings, and the o-dihydroxy structure of Ring B shows a lower antioxidative activity than the m-hydroxy structure of Ring A, which indicates the importance of the arrangement of the OH groups in the structure of the compound and that the antioxidant capacity of phenolic compounds depends on their chemical structure [85].

In clinical trials, curcumin was used in a double-blind trial in patients with amyotrophic lateral sclerosis for 6 months of treatment and decreased biomarkers of oxidative stress, including oxidative protein products, total thiols, ferric-reducing capacity, and lactate [86]. After food consumption rich in flavonoids, these are transported by serum albumin, thus modulating the bioavailability of flavonoids [87]. This bonding also allows a lower degradation of these compounds. Quercetin in oxygen-dependent processes degrades faster than when individually bound to albumin and could favor its antioxidant capacity [88]. These data indicate the potential of phenolic compounds against cell damage induced by ROS.

Clinical trials with patients with neurodegenerative diseases treated with phenolic compounds are very limited and highlight the importance of considering their application as a treatment or adjuvant to manage neurodegenerative diseases. In 2574 middle-aged adults, total and class intakes of polyphenols were evaluated and associated with their cognitive performance, which was assessed by neuropsychological tests. It was observed that general polyphenol intake was associated with better linguistic and verbal memory, while specifically, the intake of catechins, theaflavins, flavonols, and hydroxybenzoic acids was positively related to linguistic and verbal memory [89].

In a study conducted on 1367 subjects aged 65 years or older who were followed for 5 years, researchers identified, through a nutritional questionnaire, that flavonoid intake decreased the risk of dementia (RR = 0.49, 95% CI: 0.26–0.92; *p* = 0.04) [90]. In patients treated with catechin-rich oil, short-term memory improved after 1 month of intervention, and after 2 months, the ability improved further. This treatment showed neuroprotection in rats treated with nitric oxide, showing an antioxidant effect [85]. The reducing effect of polyphenols is determined by the number and arrangement of their hydroxyl groups.

Fisetin functions as an adjuvant in the treatment of individuals with ischemic stroke by extending the therapeutic window of recombinant tissue plasminogen activator (rt-PA) therapy, which is used for the management of ischemia. Patients treated with fisetin showed less impairment of cognitive abilities and decreased levels of matrix metalloproteinase (MMP) 2, MMP 9, and C-reactive protein (CRP), which are proteins involved in the healing of damaged tissue, compared to patients treated with plasminogen alone [91].

During the neurodegenerative process, the inflammation that underlies the disease is one of the contributing mechanisms in the cascade of events leading to neuronal degeneration, including microglial activation, astrogliosis, and lymphocyte infiltration [92]. Cerebral ischemia (like the neurodegenerative diseases mentioned above) is accompanied by marked inflammation initiated through the expression of cytokines and other inflammatory mediators, such as nitric oxide. Therefore, targeted therapy to attenuate inflammation has shown a significant reduction in the progression of brain damage. In treatments that induce neutropenia, the infarct volume is reduced with better recovery of the affected tissue [7].

Resveratrol is a phenol able to modulate inflammation in patients with neurodegenerative diseases. In 119 patients with mild-to-moderate AD treated with resveratrol (up to 1 g orally twice daily) for 52 weeks, levels of Aβ40, a product of Aβ precursor metabolism assessed in cerebrospinal fluid, were diminished. Resveratrol decreased the Alzheimer’s Disease Cooperative Study-Activity of Daily Living (ADCS–ADL) score during the 12-month study. It reduced the levels of IL-12P40, IL-12P70, and C-C motif chemokine ligand 5 (CCL5), demonstrating a powerful effect on regulating inflammation in the brain and suggesting the ability of phenols to cross the BBB [93].

## 9. Mechanisms of Damage Modulation by Phenolic Compounds

Glutamate is one of the multiple activators of NF-ĸB in the central nervous system through ionotropic glutamate receptors or neurotrophins [94]. The activation of NF-ĸβ in microglia during neurodegenerative processes stimulates the secretion of ROS, TNF-α, IL-1, and interferon-γ, which, in principle, attempt to remediate neurodegeneration or injury. However, continued stimulation succeeds in compromising the survival of unaffected cells. Polyphenols, including flavonoids, phenolic acids, and phenolic alcohols, can alter TLR4-signaling pathways at multiple levels. Interacting protein expression is modulated by epigallocatechin-3-gallate. Resveratrol promotes neuroprotection through TLR4-MAPK/NF-κB signaling and reduces neuronal apoptosis via the TLR4/innate immune signal-transduction adaptor (MyD88)/NF-κB pathway in microglia/macrophages [95,96]. Phenolic compounds must modulate several molecular mechanisms, such as the mitogen-activated protein kinase (MAPK)/ERK/p90 ribosomal S6 kinase (p90RSK)-signaling pathway in ischemic lesions. During inflammatory processes, they can inhibit microglial inflammation by attenuating ERK signaling and inhibiting the production of TNF-α, IL-6, IL-1β, and NO. In addition, they can increase SOD levels, promoting cell survival [97].

Resveratrol is a phenol able to inhibit the phosphorylation of PI3K, AKT, and mammalian targets of rapamycin (mTOR). The inhibition of forkhead box O (FOXO) transcription factor phosphorylation by resveratrol results in its nuclear translocation, DNA binding, transcriptional activity, increased expression of *BIM*, *TRAIL*, *DR4*, *DR5,* and *KIP1* genes, and inhibition of cyclin D1 (*CCND1*) expression. This suggests that resveratrol can regulate apoptosis (*BIM*, *TRAIL*, *DR4*, and *DR5*) and cell cycle-related genes (*KIP1* and *CCND1*) through FOXO [98]. Table 1 shows the neuroprotective effects of phenolic compounds.

**Figure 5:**
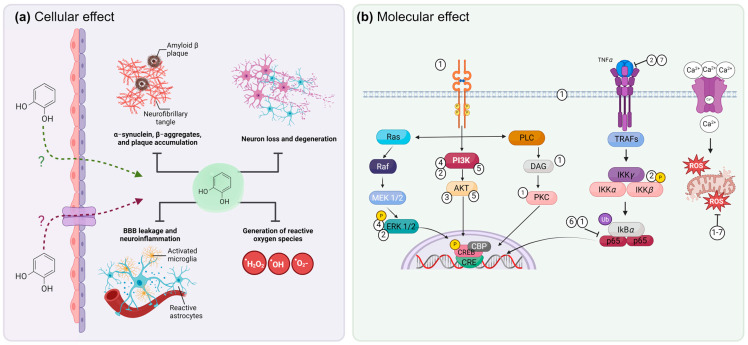
Effect of phenolic compounds on neurodegenerative processes. (**a**) The mechanism of entry of phenols has not been fully clarified, but it has been proposed that they could enter by passive diffusion (green dotted arrows), or by using some transporter (purple dotted arrows), which is still undefined. At the cellular level, phenolic compounds can prevent protein aggregate formation and neuron loss, as well as alleviate oxidative stress and neuroinflammatory processes. (**b**) Phenols favor the activation of signaling pathways involved in cell survival and the synthesis of neurotrophic factors and inhibit proteins involved in the pro-inflammatory signaling pathway (NF-kB), as well as the inhibition of ROS, an oxidizing agent of proteins, nucleic acids, and lipids, which favors the survival of neuronal populations. 1. Ferulic acid, 2. Fisetin, 3. Curcumin, 4. Resveratrol, 5. Astilbin, 6. Luteolin, 7. Genistein. CREB: cyclic adenosine monophosphate response element-binding protein; CBP: CREB: binding protein; DAG: diacylglycerol; ERK: extracellular signal-regulated kinase; IP3: inositol trisphosphate; MAPK: mitogen-activated protein kinase; MEK: mitogen-activated protein kinase kinase; PI3K: phosphatidylinositol-3-kinase; PKC: protein kinase C; PLC: phospholipase C; Trk: tropomyosin receptor kinase; TNF-α: tumor necrosis factor α; TRAF: tumor necrosis factor receptor-associated factor; IKK-γ: inhibitor of nuclear factor kappa-B kinase subunit gamma; IKK-α: inhibitor of nuclear factor kappa B kinase subunit alpha; IKK-β: inhibitor of nuclear factor kappa-B kinase subunit beta; NF-kB: nuclear factor enhancer of kappa light chains of activated B cells. The image was created in BioRender (www.biorender.com, accessed on 25 January 2024).

Using microarrays for 1200 different genes in a model of MPTP (N-methyl-4-phenyl-1,2,3,6-tetrahydropyridine) and 6-hydroxydopamine neurodegeneration, alterations were found in the expression of 49 genes involved in oxidative stress, inflammation, glutamate receptors (NMDA), and neurotrophic factors (GDNF, EGF) [60]. In addition, the analysis in knock-out mice has revealed that the deletion of certain c-Jun N-terminal kinases (JNKs) reduces the severity of neurodegenerative diseases, such as PD and cerebral ischemia [114]. JNKs are members of the MAPK family and have been shown to respond to phenolic compounds, such as resorcinol, by increasing the phosphorylation of p44/42 MAPK but not p38 MAPK [60]. This molecule is activated to a greater extent during cytotoxic cellular stress processes. Cytokines are also regulators of stress-induced apoptosis [115]. In a mouse ischemia model, the upregulation of p-ERK and p-MAPK was found, and the inhibition of ERK and MAPK phosphorylation decreased the expression of proapoptotic proteins [116]. Figure 5 shows how phenolic compounds can interact with and modify signaling pathways involved in neurodegeneration.

In mice treated with oil, palm phenolics improved cognitive function and spatial learning. Microarray gene expression analysis showed an increase in genes involved in brain development and activity, such as brain-derived neurotrophic factor (BDNF), Ca^2+^ ions and calmodulin binding, K^+^ ion transport, transmembrane receptor tyrosine phosphatase activity, and a decreased expression of genes involved in inflammation [117].

*Ginkgo biloba* derivatives were used to counteract neurodegenerative disorders in mice, where the supplementation of the extracts produced changes in the hippocampus. Changes in gene expression compared to controls, such as changes in transthyretin, AMPA-2 channel, neuronal tyrosine/phosphatase 1, and microtubule-associated τ, suggest neuroprotective functions. On the other hand, Ca^2+^, Cl^−^ channels, prolactin, and growth hormone were positively regulated. These changes were associated with improved brain functions and the activation of signaling pathways [118].

## 10. Conclusions and Perspectives

In this review, we briefly described the characteristics and some biological effects of phenolic compounds. It has been demonstrated in neurodegenerative models in vitro, in vivo, and in clinical trials that phenolic compounds can inhibit ROS, SNRs, and pro-inflammatory proteins. Phenolic compounds also regulate proteins involved in cell survival and apoptosis of neuronal cells and modulate the immune system and neuronal cell differentiation.

From this review, we suggest that phenolic compounds of low molecular weight and simple chemical structure could be a therapeutic alternative for neurodegenerative diseases, for which there is a lack of novel drugs that reduce symptoms. They would not have significant side effects compared to traditional treatment and can also be used as adjuvants. Also, treatment over long periods remains to be evaluated as antioxidants could act as pro-oxidants at high concentrations.

Promoted in vivo research and clinical trials of new phenolic compounds such as pyrogallol have been shown to have an anti-inflammatory and antioxidant effect, and some methylated metabolites can cross the blood–brain barrier more easily. In addition, it has a small size that could favor its absorption, distribution, and molecular targets related to the hallmarks of neurodegenerative diseases.

## Figures and Tables

**Table 1:** Regulation of signaling pathways by phenolic compounds.

Assay	Disease	Model	Treatment	Phenolic Compound	Finds	Ref.
In vitro	ALS	Mouse motor neuron (NSC-34) mutant hSOD1G93A gene	10 µM 4 h before the damage with H_2_O_2_	Fisetin	Fisetin reduced ROS damage, increased cell survival and the expression of phosphorylated ERK, and upregulated antioxidant factors, which were reversed by MAPK/ERK inhibition.	[99]
	AD	HT-22 Mouse Hippocampal Neuronal Cell Line	20 μM (No time specified at the same time with Aβ)	Luteolin	Decrease of inflammatory markers p-NF-kB, TNF-α, and IL-1β. Decreased proapoptotic proteins Bax, Bcl-2, Caspase-3, and Cox-2. Decrease in BACE-1 enzyme and subsequent decrease in Aβ.	[100]
	Caloric restriction associated with neurodegenerative diseases in general	Neuro2a neuroblastoma cells	10 μM (2–72 h) cells grown under serum starvation conditions	Resveratrol	Resveratrol increases AMP activation (AMPK) during caloric restriction, promoting energy conservation, cell survival, and neurite outgrowth.	[101]
	PD	PC-12 cells (6-hydroxy-dopamine) (6-OHDA)	3.1, 12.5, 50 µM or without luteolin for 2 h	Luteolin	Increased cell viability and decreased expression of proapoptotic proteins Bax and Bcl-2.	[102]
In vivo	PD	Male Wistar rats—using rotenone (ROT)-induced rat model of PD	4 weeks at the dose of 50 mg/kg, 30 min before damage is induced.	Ferulic acid	Protection of dopaminergic neurons, lipid peroxidation, and reduced levels of inflammatory markers IL-1β, IL-6, and TNF-α	[103]
	PD	(6-OHDA lesioned rats).	10 and 30 mg/kg injection 2 h before surgery and for 14 days afterward	Catechin	After treatments, they significantly reversed this abnormal motor behavior and memory deficits and protected animals from the observed decrease in dopamine and noradrenaline.	[104]
	AD	Male wildtype C57BL/6N mice	20 mg/kg/day for 2 weeks, starting 24 h after Aβ injection	Fisetin	Decreased the expression of BACE-1 and the formation of Aβ aggregates. Decreased synaptic dysfunction (promoted the expression of postsynaptic proteins PSD-95, SNAP-23, p-GluR1). Promoted activation of p-PI3K, p-AKT, and p-GSK3β and subsequent cell survival. Decreased proinflammatory proteins p-IKKβ, p-NFKB, TNFα, and IL-1β.	[105]
	PD	Male C57BL/6 mice (Parkinson’s disease model)	50 mg/kg/day for 7 days after damage with MPTP	Astilbin	Increased cell survival by promoting phosphorylation of p-PI3K/p-AKT. Decreased oxidative stress by increasing GSH and SOD activity. Reduced the loss of dopaminergic neurons and the activation of microglia (Iba-1) and astrocytes in the substantia nigra.	[106]
	Hypoxia/ischemia	Male Sprague–Dawley rats and pheochromocytoma (PC-12) cells	28, 56, and 112 mg/kg/day after ischemia for 5 consecutive days	Ferulic acid	Attenuated memory impairment reduced hippocampal neuronal apoptosis and oxidative stress in a dose-dependent manner and inactivated the Toll-like receptor TLR4 and MyD88. Increased levels of Bcl-2 and decreased levels of caspase-3 and Bax.	[107]
	TBI	Adult male ICR mice, traumatic brain injury model	50 and 100 mg/kg 30 min after TBI	Curcumin	Reduced proapoptotic proteins Bcl-2 and cleaved caspase 3. Promoted activation of the Nrf2–ARE pathway and subsequent enhancement of SOD, GPx, and MDA expression.	[108]
	Ischemic	Nine-week-old Sprague–Dawley male rats/culture primary cortical neurons	300 mg/kg 1 h after Ischemic brain injury	Curcumin	Attenuated infarction volumes, upregulated NAD(P)H, NQO1 levels. Promoted p-AKT/Nrf2 activation.	[109]
	Ischemic	Adult male C57/BL6J mice	10 mg/kg for 2 weeks before cerebral ischemia	Genistein	It decreased infarct volume, improved neurological scores, attenuated cleaved caspase-1 apoptosis, decreased the release of inflammatory factors TNF-α, IL-1β, IL-18, and IL-6, and negatively regulated inflammasome activation assessed with the NLRP3 marker.	[110]
Clinical trial/Randomized Controlled Trial	AD	Alzheimer’s disease patients	120 mg for 12 months	Genistein	The cognitive ability of the treated patients improved, and there was no increase in Aβ deposition compared to the placebo control.	[111]
	PD	Idiopathic PD patients aged ≥30	30, 80 mg/day for nine months	Curcumin	This trial was unsuccessful in showing its efficacy in quality of life and clinical symptoms of PD patients	[112]
	AD	Patients at high risk of developing Alzheimer’s disease	180 mg/day for 12 weeks	Curcumin	Reduction of circulating levels of Amyloid Polypeptide compared to the placebo control.	[113]

ERK: extracellular signal-regulated kinase, TLR: Toll-like receptor, Bcl-2: B-cell lymphoma 2, Bax: Bcl-2-associated X protein, NF-kB: nuclear factor kappa-light-chain-enhancer of activated B cells, TNF-α: tumor necrosis factor alpha, IL-1β: interleukin 1 beta, IL-6: interleukin 6, AMPK: AMP-activated protein kinase, PI3K: phosphoinositide 3-kinase, AKT: protein kinase B, GSK3β: glycogen synthase kinase 3 beta, IKKβ: inhibitor of nuclear factor kappa-B kinase subunit beta, Nrf2: nuclear factor erythroid 2-related factor 2, ARE: antioxidant responsive element, NQO1: quinone oxidoreductase 1.

## Data Availability

Not applicable.
